# Associations between Personality Traits and Basal Cortisol Responses in Sailing Athletes

**DOI:** 10.3390/ejihpe11030058

**Published:** 2021-07-24

**Authors:** Pierpaolo Limone, Maria Sinatra, Flavio Ceglie, Lucia Monacis

**Affiliations:** 1 Department of Humanities, University of Foggia, 71122 Foggia, Italy; pierpaolo.limone@unifg.it; 2 Department of Education Sciences, Psychology, Communication, University of Bari “Aldo Moro”,70122 Bari, Italy; maria.sinatra@uniba.it; 3 Department of Emergency and Organ Transplantation (DETO), Pathological Anatomy Section, University of Bari “Aldo Moro”, 70124 Bari, Italy; flceglie@libero.it

**Keywords:** sailing sport, bowmen, helmsmen, personality traits, biomarker of HPA, individual differences

## Abstract

There is a paucity of literature regarding the psycho-physiological profiles of sailors on board. This study aimed at providing empirical evidence on the individual differences between bowmen and helmsmen taking into account a biopsychological perspective. To this purpose, sailors’ profiles were examined by focusing on the association between personality traits and basal cortisol. The sample was composed of 104 athletes (M_age_ = 21.32, SD = 0.098; F = 35%), who fulfilled a self-reported questionnaire including a socio-demographic section and the Big Five questionnaire. Cortisol samples were collected on the day before the competition, within 30 min after awakening. T-test analysis showed significant differences on cortisol levels: bowmen obtained higher levels on cortisol responses compared to helmsmen. No differences emerged on personality traits between athletes’ roles. Bivariate associations showed positive associations of cortisol responses with extraversion and conscientiousness in bowmen, whereas no significant associations of cortisol with personality traits were found in helmsmen. Regression analyses confirmed that sex and extraversion predicted higher level of cortisol responses. Results were discussed in terms of a bio-psychosocial theoretical approach and provided findings on the relationships between personality trait and the hypothalamus-pituitary adrenal (HPA) system in dinghy sailors. Suggestions for a more suitable selection of sailor roles were given to coaches in order to improve athletes’ performance.

## 1. Introduction

Over the last decades, there has been a growing interest in factors improving athletes’ performance from mental abilities [1,2,3] to physiological [4], genetic [5,6], and psychological characteristics [7,8] that are decisive in winning a competition. Factors and characteristics are also valid for sailing sport [9,10,11]. Within this context, research has focused on mental skills training at any level of competition to achieve not only physical and tactical skills, but also good psychological preparation. As Pinsach and Corominas [12] claimed, sailing is peculiar and distinct from the other sports, because it takes place in an unusual (aquatic) environment, away from contact with the public, where changing atmospheric conditions demand much attention and concentration. Consequently, sailors’ exercise is based on three commands essential to performance: propulsion, steering, and balance, the latter constituting a source of emotional disturbance, primarily in inexperienced athletes [9]. Indeed, relaxation techniques and mental rehearsal are considered best practices for sailors’ health and wellbeing, against the possible occurrence of decreased concentration, nervousness, and frustration [13,14]. Even if sailing has been recognized as a sport involving psychological aspects [1,15,16,17], these aspects have been little investigated. More specifically, the associations of sailors’ performance with psychological profiles [15] and with the effects of psychological training [17] have been examined. In drawing up sailors’ psychological profiles, two levels, cognition and personality, have been generally considered. The former refers to attention, observation and anticipation capacity and to the ability not only to process a lot of information in a short time but also to develop a high strategic capacity and planning [10,11,12,13,14,15,16,17,18]. The latter is related to energy, irritability, perseverance, firmness, and resourcefulness [19].

The portrait of the psychological aspects of sailors is more complex when considering sailing as a team sports characterized by greater levels of interdependence and social interaction among athletes who play different roles on board. These two factors may in turn determine success in performance during competitions. In the categories of boats competing with two persons or more, two main roles are distinguished, bowmen and helmsmen, whose psychological profiles have been hypothesized so far only at a theoretical level: bowmen have been characterized as more extrovert and creative, whereas helmsmen have been defined as more determined and introverted with higher levels of self-control [19]. Recently, little attention has been paid to the contribution of personality to intragroup relationships and team effectiveness in sailing sport. Because of the paucity of the empirical research on this area, the current study aimed at describing the psychological profile of both sailors’ roles in order to examine individual differences in personality traits by assuming the Five Factor Model (FFM) as theoretical framework. This, in conjunction with the generic traits approach on personality and sport performance [20], show positive relations between certain traits—such as agreeableness, conscientiousness, and emotional stability—and athletic performance [21,22].

Following the trait theory of personality focused on bio-physiological correlates, the FFM identifies five broad factors: neuroticism, extraversion, openness, agreeableness, and conscientiousness [23]. The first is measured on a continuum ranging from emotional stability to emotional instability and concerns feelings of vulnerability, anxiety, and fear. People with high neuroticism scores are often over-thinking their problems and can have repercussions in terms of their relationship with others. Extraversion, measured on an introversion–extraversion continuum, refers to outgoing, socially confident behaviors. People high on this trait are full of energy, enjoy being the center of a group, and seek the attention of others, contrary to introverts, who tend to keep to themselves or maintain a close group of trusted friends. Openness to experience is related to the willingness to try new activities. People high on this trait are amenable to unconventional beliefs and to unfamiliar cultures. Conversely, low levels on this trait characterize people who are wary of uncertainty and the unknown. Agreeableness indicates the tendency to be altruistic and cooperative. Individuals with high scores on this trait often work well as members of a team, act as mediating peace-makers, and help others when needed. On the contrary, disagreeable individuals often act according to their self-interest and are more suspicious of other people’s intentions. The last trait, conscientiousness, defines those individuals who are well-organized and aware of their behaviors, show responsibility towards others and themselves carrying out the assigned duties. High levels on conscientiousness imply more goal-oriented behaviors and ambitious goals, whereas low levels connote more impulsive behaviors and a decreased interest in setting life goals. In addition, little research on team sports focused on personality differences across playing positions (offensive vs. defensive) showed inconsistent findings ranging from higher levels on extraversion in offensive position to no significant differences between the two positions [21].

Beyond the examination of the psychological characteristics linked to the role played by athletes, our research sought to analyze physiological differences in the athletes’ roles on cortisol level that is a biomarker of stress-sensitive biological systems like the hypothalamus–pituitary–adrenal (HPA) axis [24].

The current study also explored possible associations between personality traits and cortisol levels. Although the study did not directly focus on the cortisol–performance relationship, an outlook on such an association could be a starting point for improving athletes’ performance according to their specific peculiarities. Indeed, individuals can show different psychophysiological reactions to the same stressful situation, such as sport competition, relying not only on athletes’ state characteristics, but also on trait and stable characteristics. In this vein, certain traits related to more adaptive emotional regulation responses can lower the cortisol response to an acute stressor, whereas other traits facilitating higher levels of ego involvement may lead to fuel the cortisol response [25]. Consequently, certain trait characteristics, through their influence on cognitive appraisal, may have an impact on cortisol levels before, during, and after competitive situations, which could positively or negatively influence sport performance.

Concerning the association of cortisol with personality traits, mixed and inconsistent results were reported [26,27]: with neuroticism cortisol resulting to be not significant [28,29,30,31] and significant [32,33] associated; with extraversion it was found to be not significant [32,33] to significant [31,34,35,36]; with agreeableness, it resulted unrelated [36,37,38] or related to [34,39]; likewise, with openness unrelated [35,40] and significant related to [30,34,38]; finally, with conscientiousness unrelated [31,41] or significant associated [35,42].

### Main Hypotheses

In line with the aforementioned theoretical assumptions of psychological characteristics of the personality traits, we expected significant differences in mean scores in certain personality traits related to the two roles played on board: like athletes playing in defensive position, helmsmen would score high on conscientiousness, since they are more prone to use problem-focused coping strategies when they maneuver the boat in all environmental conditions and critical situations (H1); like athletes playing in offensive position, bowmen would score high on extraversion (H2), since they control sails and spinnakers. Given the lack of evidence or competing findings from previous studies, we were agnostic about the differences in remaining traits. Indeed, no other differences in personality traits were hypothesized.

As for the examination of physiological differences in the athletes’ roles, we expected higher mean scores of cortisol levels in bowmen, since they generally experience a higher degree of physical stress on board (H3).

Finally, on basis of these above-mentioned compelling findings on the association of cortisol with personality traits and following Kern and Friedman’s assumption [43] that extroverts are characterized by a “biologically-based drive for activity”, we hypothesized a significant effect of extraversion on basal cortisol in bowmen (H4), since they are more oriented to the surrounding environments on board and are more subjected to physical stress experienced in short time during physical maneuvers. Given the merely descriptive nature of our study, no effects of other traits on cortisol were hypothesized.

## 2. Materials and Methods

### 2.1. Participants

The sample was composed of 104 sailors (35% females; M*_age_* = 21.32, SD = 0.098; 58% bowmen, M*_age_* = 20.80, SD = 0.097, and 42% helmsmen, M*_age_* = 22.12, SD = 0.099). All athletes with a sport experience of 6.85 years (SD = 2.237) were engaged in the Italian Youth Two Crew Members Dinghy Classes Championship. All athletes came from Italian regions.

### 2.2. Procedure 

Written informed consent was obtained from the participants. The research proposal was approved by the Committee for Research Ethics of the local University (No. 0045912) and was conducted in accordance with the Helsinki declaration and the ethical rules of the Italian Psychological Association. A self-reported survey including information on gender, age, and sailors’ role on board and the psychological assessment was voluntary completed by athletes on the day before the national regatta (held in early September 2020) in Southern Italy.

### 2.3. Instruments

#### 2.3.1. Salivary Cortisol Assay

The saliva specimens were collected by participants under coaches’ supervision on the day before the competition within 30 min after awakening (from 7am to 8 am), and using cotton swabs and saliva collecting tubes (Salivette, Sarstedt, Germany). The cotton swab was placed into mouth for 2 min and participants were instructed to chew 20 times. The saliva collecting tubes were centrifuged at 3000 rev/min for 15 min at 4 °C and stored at −80°C until they were assayed and tested in the same series to avoid any variations between tests. An enzyme immunoassay kit (Salimetrics, LLC, Carlsbad, CA, USA) was used to measure salivary-free cortisol concentrations and according to the manufacturer’s instructions. Intra-assay coefficient of variation of 3.5 ± 0.5% and an inter-assay reproducibility of 5.08 ± 1.33% were accepted, whose levels were expressed in no/L.

#### 2.3.2. Personality Traits

The validated Italian version of the Big Five Questionnaire-2 (BFQ-2) [44] was used to assess sailors’ personality traits. It comprises 134 items that describe five dimensions (extroversion, agreeableness, conscientiousness, neuroticism, openness to experience) ranging on a 5-point Likert-type scale from complete disagreement (very false for me) to complete agreement (very true for me). Each dimension is assessed by means of 24 items. The instrument also provides a Lie scale (14 items), which measures socially desirable responding. In the current research, fit indices obtained from confirmatory factor analysis were adequate, χ2 = 329.14, df =200, *p* < 0.000, CFI = 0.951, RMSEA = 0.070 (95% confidence interval (CI) = 0.064, 0.080), SRMR = 0.029 and the internal consistency values of the five traits ranged from 0.82 to 0.85.

### 2.4. Statistical Analysis

The normality of the data was checked. The Shapiro–Wilk test indicated a normal distribution of the data, since its *p*-values for each variables were larger than 0.05. Specifically, the test was equal to 0.386 (helmsmen) and 0.702 (bowmen) for cortisol levels, to 0.493 (helmsmen) and 0.691 (bowmen) for extraversion, to 0.433 (helmsmen) and 0.424 (bowmen) for agreeableness, 0.276 (helmsmen) and 0.796 (bowmen) for conscientiousness, to 0.207 (helmsmen) and 0.943 (bowmen) for neuroticism, and 0.254 (helmsmen) and 0.333 (bowmen) for openness to experience. Moreover, the normality was also confirmed by Kolmogorov–Smirnov test (even if it used in the case of a large sample) given that all *p*-values associated were not significant (*p* > 0.05). A visual inspection of the histograms and box plots showed that cortisol levels and the mean scores of each personality traits were approximately normally distributed for both helmsmen and bowmen. Finally, values of kurtosis and skewness of all variables were within the range of ± 1.960 and are reported in Table 1.

Descriptive statistics and zero-order correlations between the variables of interest were applied to the total sample and to sailor roles. Independent samples t-test was run to examine the role differences among the variables of interest. Regression analysis was performed to identify which of the personality trait was associated with the levels of cortisol. We used the stepwise method in procedure of multiple regression analysis, because it was the most appropriate way to determine the association between variables. All analyses (independent t-test, correlation, and regression) were run with 95% bias-corrected and accelerated (BCa) confidence intervals (bootstrap sample of 2000).

## 3. Results

Table 2 shows descriptive statistics related to mean and standard deviation for each variable in the total sample and in the role category. Results from the t-test indicated no significant effects of the role on the five traits, i.e., on extraversion [t(102) = 0.632, *p* > 0.050], agreeableness [t(102) = −0.113, *p* > 0.050], conscientiousness [t(102) = −1.056, *p* > 0.050], neuroticism [t(102) = −0.956, *p* > 0.050], and openness to experience [t(102) = −0.527, *p* > 0.050], but a significant effect of the role on cortisol level [t(102) = −2.065, *p* < 0.050]. Bowmen obtained higher mean scores on cortisol levels in comparison to helmsmen. Moreover, according to Cohen’s suggestions, the effect size was considered medium, thus implying that the difference of means from two groups was medium enough to be really important.

Table 3 shows bivariate correlations coefficients among the variables of interest in the total sample and in the sailing role category. Findings indicated no significant association between cortisol levels and personality traits in the total sample, whereas positive associations of cortisol level with extraversion and conscientiousness were found in the bowmen group.

On the basis of the correlation results, hierarchical multiple regression was conducted only for the bowmen category. Gender was entered at stage one, extraversion at stage two and conscientiousness at stage three. The hierarchical multiple regression revealed that in Model 1 gender did not contribute significantly to the regression model, F(1,58) = 3,905, *p* > 0.05, β = −0.481. In Model 2, the trait of extraversion explained an additional 24.6% of variation in cortisol level, and this change in R² was significant, F(2,56) = 5.477, *p* < 0.05, β = 0.497, *p* < 0.05. Gender became significant, β = −0.459, *p* < 0.05. In Model 3, the trait conscientiousness explained an additional 11% of the variation in cortisol level, although this change in R² was not significant, F(3,54) = 2.948, *p* > 0.05. Therefore, the last trait did not account for a significant amount of variance above and beyond extraversion. Table 4 reports regression statistics. To sum up, our findings showed that being male with high scores on extraversion is associated with higher levels of cortisol response.

## 4. Discussion

In spite of the increasing interest in analyzing those psychological factors that may enhance sailors’ performance, there is a lack of empirical research on individual differences between the roles played by athletes on board, i.e., helmsmen and bowmen. To fulfill this gap, we examined the differences in personality traits and in cortisol levels in order to draw up the psycho-physiological portrait of these athletes. Being cortisol a neuroendocrine manifestation of the HPA axis sensitive to psychological stressors, its inspection can be useful to best identify the specific characteristics related to the two roles. Findings from t-test analysis did not support H1 and H2, since no significant difference was found in personality traits between helmsman and bowmen. Furthermore, a careful inspection of the mean scores, albeit not significant, revealed an opposite direction of the expected values: higher mean scores on conscientiousness hypothesized in helmsmen were obtained in bowmen; higher mean scores on extraversion hypothesized in bowmen were obtained in helmsmen, Therefore, the distinction which was theoretically developed by Manzanares Serrano and colleagues [19] and formulated on the basis of defensive and offensive positions generally played by athletes in team sports was not empirically supported within the Five Factor Model, and results also revealed opposite mean scores in each trait dimension.

By contrast, H3 was confirmed given the emerged different mean levels of cortisol between the two categories. As expected, higher levels of cortisol were found in bowmen in comparison with their counterpart: in line with Kern and Friedman’s [43] bio-psychosocial perspective implying a “biologically-based drive for activity”, this finding might corroborate bowmen’ more action-oriented behaviors. In addition and consistently with this last perspective, results confirmed the significant effect of extraversion on cortisol in bowmen, thus supporting H4. Findings from regression analyses indicated that male bowmen with higher scores on extraversion tend to show higher cortisol levels. This is in line with studies reporting such significant relationships [30,42] and in contrast with research showing no associations [31,32]. A further interesting observation concerned gender: contrary to prior studies, providing a significant association between high extraversion and cortisol levels in females [28,35], this association was found only in males. This unexpected finding may be due to the unbalanced and limited female sample size. Further investigations need to be carried out to clarify this issue.

Finally, bivariate correlation showed a positive association between cortisol and conscientiousness in bowmen, thus implying that bowmen who are more prone to show more reflective behaviors and an increased interest in setting life goals, seem to be also characterized by high levels of *mental* stress. In other terms, they seem to be twice as likely to face the risk of stress, mental and physical, than their counterpart. This result was consistent with Laucelle et al.’s research [42]. Beyond the significant associations found in bowmen, no relationships of cortisol with other personality traits were proved to be significant.

## 5. Conclusions

This descriptive picture provided a modest empirical insight into the conflicting and mixed results previously showed in scientific literature.

Some practical suggestions could be inferred from our research. In order to put sailors in positions where they can use strengths and abilities, coaches should not only enhance mental skills, such as cognitive, emotional, and behavioral strategies, but also they should keep in mind there are psycho-biological correlates, such as personality traits and biomarkers, that should be taken into account simultaneously as key factors in identifying the best sailing role played on board. In particular, the linkage of personality characteristics with basal cortisol levels may indicate how bowmen and helmsmen should be selected according to their psycho-physiological dispositions in order to adapt specific trainings.

The findings may also indicate that baseline information of psycho-physiological variables could be beneficial in order to target support for athletes at higher potential risk for poor performance and stress during a sport competition. Future studies should investigate appropriate mental trainings, like mindfulness-based interventions [45] or relaxation/meditation programs [9], on the basis of individual dispositions related to the role played by athletes on board. Such interventions may be applied to enhance a good psychological preparation and thus, a performance improvement.

Our study suffers from some limitations. First, the cortisol data collection was limited to a single awakening time, and, therefore, no changes in response to a stressor or in diurnal variations were considered. Consequently, a broader comparison with prior findings was restricted. Second, the correlational nature of the results implied that causal relationships could not be inferred. Moreover, as previously affirmed, the sample size was modest (just over 100 participants) and was affected by gender-bias, thus decreasing the statistical power. Third, given the exploratory nature of this investigation, the examination of the effects of individual differences in personality traits and in cortisol concentrations on sport performance was not analyzed. Nevertheless, this research also has the strength to explore for the first time the associations between personality traits and cortisol levels among sailors. Further studies are recommended to deepen the knowledge of individual differences of sailors in relation to their role on board and to performance in competitions.

## Figures and Tables

**Table 1:** Descriptive statistics of values of skewness and kurtosis for each variable.

Variables	Helmsmen	Bowmen	*Z-Values*
Cortisol			
Skewness	−0.271	−0.150	1.799
SE	0.464	0.580	0.799
Kurtosis	0.247	−0.894	−0.276
SE	0.902	1.121	0.804
Extraversion			
Skewness	0.396	0.275	1.438
SE	0.464	0.580	0.799
Kurtosis	−0.587	0.595	−0.988
SE	0.902	1.121	0.804
Agreeableness			
Skewness	−0.005	−0.451	0.011
SE	0.464	0.580	0.799
Kurtosis	−0.811	−0.461	1.761
SE	0.902	1.121	0.804
Conscientiousness			
Skewness	0.585	−0.339	−1.728
SE	0.464	0.580	0.799
Kurtosis	0.301	−0.176	−1.710
SE	0.902	1.121	0.804
Neuroticism			
Skewness	−0.750	−0.394	1.906
SE	0.464	0.580	0.799
Kurtosis	0.320	−0.551	−0.580
SE	0.902	1.121	0.804
Openness			
Skewness	0.458	0.420	1.091
SE	0.464	0.580	0.799
Kurtosis	−0.353	−0.945	0.373
SE	0.902	1.121	0.804

**Table 2:** Descriptive statistics: Mean and standard deviation for each variable in the total sample, in helmsmen and bowmen.

Sample	Cortisol Level	Extraversion	Agreeableness	Conscientiousness	Neuroticism	Openness
Total sample	3.93 (1.24)	83.96 (12.63)	84.13 (11.81)	83.34 (11.22)	65.88 (14.64)	81.12 (12.67)
Helmsmen	3.63 (0.93)	82.50 (12.87)	84.67 (10.24)	82.76 (11.20)	64.16 (14.20)	80.40 (12.05)
Bowmen	4.43 (1.53)	81.05 (12.45)	85.07 (14.44)	84.97 (11.22)	68.73 (15.42)	82.36 (13.98)
t-test (95%CI)	−2.065 * [−1.611; −0.038]	0.632 [−5.180; 10.312]	−0.113 [−8.112; 7.658]	−1.056 [−10.649; 2.818]	−0.956 [−13.820; 4.901]	−0.527 [−11.427; 6.735]
Cohen’s d	0.63	-	-	-	-	-

* *p* < 0.050.

**Table 3:** Bivariate correlations between personality traits and levels of cortisol in the total sample, in helmsmen and bowmen.

Cortisol	Extraversion	Agreeableness	Conscientiousness	Neuroticism	Openness
Total sample					
Cortisol Level	0.212	0.142	0.245	0.184	0.215
(95%CI)	[−0.089; 0.466]	−0.132; 0.452]	[−0.049; 0.532]	[−0.108; 0.423]	[−0.113; 0.494]
Helmsmen					
Cortisol Level(95%CI)	0.019 [ −0.269; 0.274]	0.091 [−0.282; 0.357]	−0.116 [−0.495; 0.190]	−0.173 [−0.547; 0.242]	0.157 [−0.170; 0.381]
Bowmen					
Cortisol Level(95%CI)	0.580 * [0.014; 0.892]	0.239 [−0.235; 0.838]	0.536 * [0.065; 0.862]	0.421 [−0.075; 0.786]	0.256 [−0.450; 0.763]

* *p* < 0.050.

**Table 4:** Summary of Hierarchical Regression Analysis for Variables predicting Cortisol Level.

Model	*Β*	t	R	R^2^	ΔR^2^
Step 1			0.482	0.231	0.231
Gender	−0.481	−1.876			
Step 2			0.691	0.477	0.246 *
Gender	−0.459	−2.198 *			
Extraversion	0.497	2.378 *			
Step 3			0.767	0.588	0.110
Gender	−0.368	−1.829			
Extraversion	0.365	1.886			
Conscientiousness	0.364	1.717			

* *p* < 0.050.

## Data Availability

The data are available on request from the corresponding author.

## References

[1] Brandt R., Da Silveira Viana M., Brusque Crocetta T., Andrade A. (2016). Association between mood states and performance of Brazilian elite sailors: Winners vs. non-winners. Cult. Cienc. Y Deporte.

[2] Manzanares A., Menayo R., Segado F., Salmerón D., Cano J.A. (2015). A probabilistic model for analysing the effect of performance levels on visual behaviour patterns of young sailors in simulated navigation. Eur. J. Sport Sci..

[3] Monacis L., De Palo V., Sinatra M. (2015). Motivational factors related to aggression within martial arts context. Rev. De Psicol. Del Deporte.

[4] Sanhueza J.A., Zambrano T., Bahamondes-Avila C., Salazar L.A. (2016). Association of anxiety-related polymorphisms with sports performance in Chilean long distance triathletes: A pilot study. J. Sport Sci. Med..

[5] Guilherme J.P.L.F., Bigliassi M., Lancha Junior A.H. (2019). Association study of SLC6A2 gene Thr99Ile variant (rs1805065) with athletic status in the Brazilian population. Gene.

[6] Peplonska B., Safranow K., Adamczyk J. (2019). Association of serotoninergic pathway gene variants with elite athletic status in the Polish population. J. Sports Sci..

[7] Resch M. (2010). The psychological factors affecting athletic performance. Orv. Hetil..

[8] Monacis L., De Palo V., Sinatra M. (2014). Sportspersonship behaviours: An exploratory investigation of antecedents. Int. J. Sport Psychol..

[9] Allen J.B., De Jong M.R. (2006). Sailing and sports medicine: A literature review. Br. J. Sports Med..

[10] Caraballo I., González-Montesinos J.L., Alías A. (2019). Performance factors in dinghy sailing: Laser class. Int. J. Environ Res..

[11] Sagayama H., Toguchi M., Yasukata J., Yonaha K., Higaki Y., Tanaka H. (2019). Total energy expenditure, physical activity level, and water turnover of collegiate dinghy sailors in a training camp. Int. J. Sport Nutr. Exerc. Metab..

[12] Pinsach J., Corominas J. (2002). Entrenamiento psicológico en vela.

[13] Maynard I., Dosil J. (2006). The sport psychology of Olympic sailing and windsurfing. The Sport Psychologist’s Handbook: A Guide for Sport-Specific Performance Enhancement.

[14] Segato L., Brandt R., de Liz C.M., Vasconcellos D., Andrade A. (2010). Psychological stress in high level sailors during competition/Estresse psicologico de velejadores de alto nivel esportivo em competicao. Motricidade.

[15] Fernandes H.M., Bombas C., Lázaro J.P., Vasconcelos-Raposo J. (2007). Perfil psicológico e sua importância no rendimento em vela. Motricidade.

[16] Martorell M.S., Ponseti F.J., Prats A.N., Bosch B.C., Ferragut G.S., García-Mas A., Prats A.N. (2021). Competitive anxiety and performance in competing sailors [Ansiedad competitiva y rendimiento en deportistas de vela]. Retos.

[17] Olmedilla A., Ortega E., González J., Serpa S. (2015). Psychological training in sailing: Performance improvement for the Olympic classification phase. Univers. J. Psychol..

[18] Morales-Belando M.T., Arias-Estero J.L. (2017). Influence of teaching games for understanding on game performance, knowledge, and variables related to adherence in youth sailing. J. Teach Phys. Educ..

[19] Manzanares Serrano A., Segado Segado F., Menayo Antúnez R. (2012). Factores determinantes del rendimiento en vela deportiva: Revisión de la literatura. Cult. Cienc. Y Deporte.

[20] De Vries R.E. (2020). The main dimensions of sport personality traits: A lexical approach. Front. Psychol..

[21] Allen M.S., Greenlees I., Jones M. (2013). Personality in sport: A comprehensive review. Int. Rev. Sport Exerc. Psychol..

[22] Steca P., Baretta D., Greco A., D’Addario M., Monzani D. (2018). Associations between personality, sports participation and athletic success. A comparison of Big Five in sporting and non-sporting adults. Pers. Individ. Differ..

[23] Costa P.T., McCrae R.R. (1992). The five-factor model of personality and its relevance to personality disorders. J. Pers. Disord..

[24] Anderson T., Wideman L. (2017). Exercise and the cortisol awakening response: A systematic review. J. Sports Med..

[25] Lautenbach F., Laborde S., Raab M., Lobinger B., Hoffman S., Pizzera A., Laborde S. (2016). The influence of hormonal stress on performance. Performance Psychology.

[26] Soliemanifar O., Soleymanifar A., Afrisham R. (2018). Relationship between personality and biological reactivity to stress: A review. Psychiatry Investig..

[27] Sundin Z.W., Chopik W.J., Welker K.M., Ascigil E., Brandes C.M., Chin K., Ketay S., Knight E.L., Kordsmeyer T.L., McLarney-Vesotski A.R. (2021). Estimating the Associations between Big Five Personality Traits, Testosterone, and Cortisol. Adapt. Hum. Behav. Physiol..

[28] Hill E.M., Billington R., Krägeloh C. (2013). The cortisol awakening response and the big five personality dimensions. Pers. Individ. Differ..

[29] Ormel J., Bastiaansen A., Riese H., Bos E.H., Servaas M., Ellenbogen M., Rosmalen J.G., Aleman A. (2013). The biological and psychological basis of neuroticism: Current status and future directions. Neurosci. Biobehav. Rev..

[30] Ouanes S., Castelao E., von Gunten A., Vidal M., Preisig M., Popp J. (2017). Personality, cortisol, and cognition in non-demented elderly subjects: Results from a population-based study. Front. Aging Neurosci..

[31] van Santen A., Vreeburg S.A., Van der Does A.W., Spinhoven P., Zitman F.G., Penninx B.W. (2011). Psychological traits and the cortisol awakening response: Results from the Netherlands Study of Depression and Anxiety. Psychoneuroendocrinology.

[32] Bibbey A., Carroll D., Roseboom T.J., Phillips A.C., de Rooij S.R. (2013). Personality and physiological reactions to acute psychological stress. Int. J. Psychophysiol..

[33] Mangold D., Mintz J., Javors M., Marino E. (2012). Neuroticism, acculturation and the cortisol awakening response in Mexican American adults. Horm. Behav..

[34] Montoliu T., Hidalgo V., Salvador A. (2020). Personality and Hypothalamic–Pituitary–Adrenal Axis in Older Men and Women. Front. Psychol..

[35] Nater U.M., Hoppmann C., Klumb P.L. (2010). Neuroticism and conscientiousness are associated with cortisol diurnal profiles in adults-role of positive and negative affect. Psychoneuroendocrinology.

[36] Munafò M.R., Lee L., Ayres R., Flint J., Goodwin G., Harmer C.J. (2006). Early morning salivary cortisol is not associated with extraversion. Pers. Individ. Differ..

[37] Zobel A., Barkow K., Schulze-Rauschenbach S., Von Widdern O., Metten M., Pfeiffer U., Schnell S., Wagner M., Maier W. (2004). High neuroticism and depressive temperament are associated with dysfunctional regulation of the hypothalamic-pituitary-adrenocortical system in healthy volunteers. Acta Psychiatr. Scand..

[38] LeBlanc J., Ducharme M.B. (2005). Influence of personality traits on plasma levels of cortisol and cholesterol. Physiol. Behav..

[39] Oswald L., Zandi P., Nestadt G., Potash J.B., E Kalaydjian A., Wand G.S. (2006). Relationship between Cortisol Responses to Stress and Personality. Neuropsychopharmacology.

[40] Wirtz P.H., Elsenbruch S., Emini L., Rüdisüli K., Groessbauer S., Ehlert U. (2007). Perfectionism and the cortisol response to psychosocial stress in men. Psychosom. Med..

[41] Parent-Lamarche A., Marchand A. (2015). The moderating role of personality traits in the relationship between work and salivary cortisol: A crosssectional study of 401 employees in 34 Canadian companies. BMC Psychol..

[42] Schoofs D., Hartmann R., Wolf O.T. (2008). Neuroendocrine stress responses to an oral academic examination: No strong influence of sex, repeated participation and personality traits. Stress.

[43] Laceulle O.M., Nederhof E., van Aken M.A.G., Ormel J. (2015). Adolescent personality: Associations with basal, awakening, and stressinduced cortisol responses. J. Pers..

[44] Kern L.M., Friedman H.S. (2011). Personality and pathways of influence on physical health. Soc. Personal. Psychol. Compass.

[45] Caprara G.V., Barbaranelli C., Borgognoni L., Vecchione M. (2007). BFQ-2 Big Five Questionnaire–2 Manuale.

